# Activated Carbon-Supported Pt Catalysts Intended for the Hydroprocessing of Lipid Feedstocks: Effects of Support Surface Composition and Impregnation Protocol

**DOI:** 10.3390/molecules30132862

**Published:** 2025-07-04

**Authors:** Ruana D. Brandão, Antônio M. de Freitas Júnior, José J. Linares, Paulo A. Z. Suarez, Romulo C. Dutra, Jeremie Garnier, Myller S. Tonhá, Daniel Ballesteros-Plata, Enrique Rodríguez-Castellón, Marcos J. Prauchner

**Affiliations:** 1 Institute of Chemistry, University of Brasilia, Campus Darcy Ribeiro, Brasilia CEP 70904-970, DF, Brazil; ruanabrandao@gmail.com (R.D.B.); antoniomartinsdefreitasjunior@gmail.com (A.M.d.F.J.); joselinares@unb.br (J.J.L.); psuarez@unb.br (P.A.Z.S.); romulodutrac@gmail.com (R.C.D.); 2 Department of Academic Areas, Federal Institute of Goiás, Rua 54, esq. com Rua 11, Parque Lago, Formosa CEP 73813-816, GO, Brazil; 3 Federal Institute of the North of Minas Gerais, Campus Arinos, Arinos CEP 38680-000, MG, Brazil; 4 Institute of Geosciences, University of Brasilia, Campus Darcy Ribeiro, Brasilia CEP 70910-900, DF, Brazil; garnier@unb.br (J.G.); myller@unb.br (M.S.T.); 5 Department of Inorganic Chemistry, Crystallography and Mineralogy, Inter-University Institute of Research in Biorefineries I3B, Faculty of Sciences, University of Málaga, 29071 Malaga, Spain; daniel.ballesteros@uma.es

**Keywords:** hydrocarbon biofuels, jet biofuel, HEFA, HDO, Pt/AC, activated carbon, catalysis

## Abstract

This work concerns the preparation of Pt/AC catalysts (Pt supported on activated carbon) and their application to the synthesis of hydrocarbon biofuels through the HEFA (hydroprocessing of esters and fatty acids) route. The key motivation for the work was that catalysts based on sulfided Mo supported on γ-Al_2_O_3_, traditionally employed in the hydroprocessing of petroleum derivatives, (i) are unstable in the HDO (hydrodeoxygenation) of biomass-derived feedstocks and (ii) can contaminate the resulting biofuels with sulfur. In this context, a systematic study on the effects of preparation conditions on the properties of the resulting Pt/AC catalysts and their performance in HEFA was carried out for the first time. Efficient catalysts were obtained, which led to the complete deoxygenation of lauric acid and coconut oil, yielding products composed primarily of *n*-alkanes. The highest HDO activity was verified for the catalyst prepared using as a support an AC previously subjected to thermal treatment up to 800 °C in a H_2_ atmosphere (which removed most of the surface acidic oxygenated groups), depositing Pt over the surface of this support via wet impregnation using a H_2_PtCl_6_ solution acidified with HCl. The obtained results showed the great potential of the Pt/AC catalysts for the production of hydrocarbon biofuels through the HEFA route.

## 1. Introduction

Intensive global efforts have been devoted to developing hydrocarbon biofuels, which offer a compelling alternative for diversifying the world’s energy matrix and making it more sustainable. Hydrocarbon biofuels exhibit properties and performance akin to those of petroleum fuels, can utilize existing supply infrastructure (tanks, pipelines, pumps, etc.), can be used without engine modification, and can be blended with conventional fuels in any proportion, thus complying with the “drop-in” concept. These issues are particularly relevant for producing SAF (sustainable aviation fuel), given the risks inherent to the aviation sector. However, diesel-like hydrocarbon production has also increased [1,2,3].

Currently, HEFA (hydroprocessing of esters and fatty acids) is the most advanced technology available to produce hydrocarbon biofuels. The key step in HEFA is the deoxygenation of fatty chains resulting from the split of acylglycerides that constitute lipid feedstocks (vegetable, animal, and algal oils and fats). This is achieved through a process similar to that employed in the hydroprocessing of petroleum and its derivatives. i.e., thermal treatment at temperatures between around 300 and 400 °C, under elevated H_2_ pressure (~20–100 bar), and in the presence of a heterogeneous catalyst [1,2,3,4,5,6]. This procedure is usually called HDO (hydrodeoxygenation). The possibility of retrofitting existing petroleum refinery facilities to perform HEFA is a major advantage of this process.

The HDO of lipid feedstocks and other O-rich mixtures (e.g., lignocellulose-derived bio-oils) can be carried out in the presence of the same catalysts employed in the hydroprocessing of petroleum and its derivatives, i.e., sulfides of group 6 transition metals (Mo or W), promoted by a transition metal with a higher number of d electrons (Ni or Co), deposited on porous oxide supports (mainly γ-Al_2_O_3_) [4,5,6,7,8,9]. However, some published research has stressed that these catalysts are not ideal for hydroprocessing O-rich mixtures. From a support perspective, challenges are presented by the fact that inorganic materials such as γ-Al_2_O_3_ are susceptible to hydrolysis by the abundant water formed during the process, which can lead to catalyst deactivation [10]. In turn, from the perspective of the metal-containing phase, the fact that the formed water (and possibly other oxygenated molecules such as CO_2_) causes sulfur replacement by oxygen, which gradually reduces the HDO catalyst activity [4,11,12,13,14,15,16], presents an additional challenge. To overcome this problem, some authors have added sulfiding agents like dimethyl disulfide (DMDS) or H_2_S [4,11,12,13,14,15,16,17] alongside the main feed. Another reported issue with using sulfided catalysts in HDO is their potential to contaminate the produced biofuel with sulfur. This contamination can originate from catalyst leaching or the co-fed sulfiding agents [13,14,15,18]. This is particularly concerning given that, unlike petroleum derivatives, biofuels are naturally nearly sulfur-free.

In this scenario, the present work aimed to develop sulfur-free heterogeneous catalysts with improved activity and stability for the HDO of lipid feedstocks. To reach this goal, metallic Pt was used as the active phase, supported by homemade activated carbons (ACs).

The choice to use ACs as a support was mainly justified by their high thermal and chemical stability in both acidic and basic media, including resistance to hydrolysis [18,19,20]. Furthermore, ACs exhibit low acidity, which contributes to reduce hydrocracking and coke formation [19,21,22]; present larger surfaces areas than those of inorganic supports; display a pore size distribution and surface chemistry that can be tailored according to the envisaged application [23,24,25,26]; can be obtained from cheap and abundant precursors such as biomass residues, coal, and petroleum coke; facilitate the recovery of the metallic phase from spent catalysts by burning the carbon [18,19]; can be produced in various forms, such as powder, grains, fibers, pellets and monoliths, providing great versatility [26,27,28]; and present good mechanical properties, depending on the precursor and the employed activation methodology and conditions [25,26,29].

Concerning the metallic phase, Pt is well-known for its ability to activate a wide range of reactions, including hydrogenation, dehydrogenation, hydrogenolysis, and oxygen electroreduction. Pt supported on the surface of acidic porous solids has been employed to promote the hydroisomerization of *n*-alkanes into iso-alkanes [30,31,32,33], which allows for improvement in the cold properties of hydrocarbon fuels, making them more appropriate for use in the aviation sector [1]. Furthermore, Pt deposited on different inorganic supports (e.g., γ-Al_2_O_3_, SiO_2_, Nb_2_O_5_, ZrO_2_, TiO_2_, and several zeolites) has been used to promote HDO, as reported in several reviews [2,3,4,5,6,11,12,13,34,35,36]. Notably, Chen et al. [37] employed Pt deposited on SAPO-11 zeolite to promote the hydroconversion of Jatropha oil into a hydrocarbon mixture with a high iso-alkane content (HDO and hydroisomerization took place in a one-step process).

Nevertheless, the literature contains few studies regarding the use of Pt supported on ACs (Pt/ACs) in the HDO of lipid feedstocks. Among these few works, Jin et al. [38] reported a high conversion of palm oil into alkanes (>90%) during hydroprocessing in a fixed-bed reactor (T = 300 °C, 30 bar of H_2_, and LHSV = 1.5 h^−1^) over Pt (1 wt%) supported on the surface of a N-doped AC. In addition to excellent performance, the catalyst showed notable stability. The authors concluded that the nitrogen incorporated into the carbon favored the interaction with carbonyls, thereby boosting decarboxylation reactions. In turn, Makcharoen et al. [39] investigated the effects of various operating parameters on the hydroprocessing of crude palm kernel oil (CPKO) over an AC-supported Pt catalyst (5 wt% of Pt) in a fixed-bed reactor. The best result, with a yield of 58.3% in biojet fuel, was obtained at operating conditions of 420 °C, 34.5 bar, with a H_2_ flow of 17.5 mL min^−1^, a CPKO flow of 0.02 mL min^−1^, and a H_2_-to-oil molar ratio of 28.0.

At this point, it is worth mentioning that the high cost of Pt demands the optimization of its catalytic activity. However, despite extensive research on the use of Pt/AC catalysts across a large range of applications, there are currently no definitive conclusions regarding the methods and conditions that yield optimal catalytic activity, which can be attributed to the complexity of the system and the large number of variables to be considered. In general, high catalyst activity is considered to be directly related to a high dispersion of the metallic phase on the surface of a solid with appropriate porosity. The dispersion depends on factors such as the properties of the support (surface area and surface chemistry), the nature of the metal precursor, the impregnation procedure employed, the polarity of the solvent, and the pH of the impregnating solution. In turn, appropriate porosity results in a pore size distribution that not only provides a high surface area for metal dispersion but also ensures the accessibility of the reactants and products to and from the catalytic active sites within the pore network.

The described scenario sparked our interest in further investigating the use of Pt/AC catalysts in the HDO of lipid feedstocks, with a particular focus on identifying the optimal conditions for obtaining catalysts with improved performance. We investigated the influence of the support surface chemistry (ACs with varying contents of oxygenated functional groups were synthesized and employed), the impregnation methodology (incipient wetness impregnation versus wet impregnation), and the acidification of the impregnating solution with HCl.

## 2. Results

### 2.1. AC Supports

Three ACs with varying surface chemical compositions were employed as supports in the Pt-based catalysts prepared in this work: P54, P54Red, and P54Ox. P54 was synthesized by the chemical activation of a dried endocarp of coconut shell with H_3_PO_4_, as detailed in Section 3.1. This activation methodology was chosen because (i) compared to other methods (i.e., physical activation and chemical activation with KOH), it favors the obtaining of mesopore-rich materials, provided that a relatively high phosphorus/shell ratio is employed [40]; (ii) chemical activation of hard lignocellulosic raw materials (as is the case with dried endocarp of coconut shell) with H_3_PO_4_ typically yields ACs with improved mechanical properties, as addressed elsewhere [26,40]. At this point, it is important to emphasize that mesopore-rich supports are highly advantageous in catalysts meant for use in liquid phase processes because large pores enhance the accessibility of the reactants and products to and from the catalytic active sites within the pore network [11,41,42]. In this work, a phosphorus/shell mass ratio of 0.54 was used (for this reason, the resulting AC was termed P54).

Two additional AC supports, P54Red and P54Ox, were prepared by modifying P54 through (i) thermal treatment up to 800 °C in a reductive H_2_ atmosphere, which aimed to remove functional groups from the AC surface; (ii) refluxing with an oxidative HNO_3_ solution, which aimed to increase the content of oxygenated functional groups on the AC surface (the treatments are described in Section 3.2). This allowed for the investigation of the influence of the support surface chemistry on the properties of the obtained catalysts.

The characterization of P54 is presented in Section 2.1.1. In turn, the changes promoted by the reductive treatment in a H_2_ atmosphere and the oxidative treatment with HNO_3_ are depicted in Section 2.1.2 and Section 2.1.3, respectively.

#### 2.1.1. Unmodified AC P54

**Figure 1:**
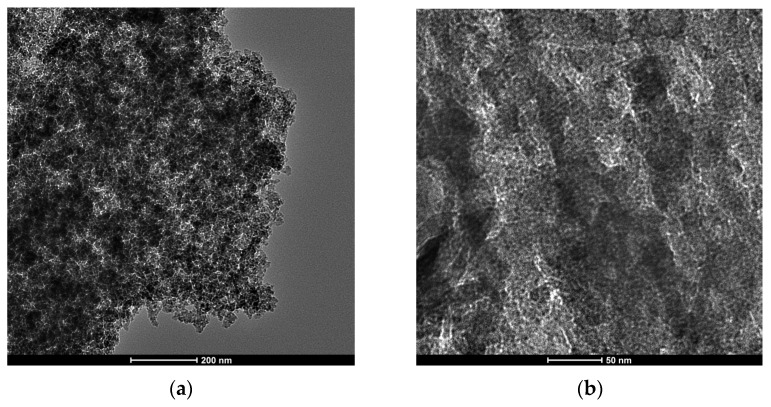
TEM micrographs of the bare AC P54 at (**a**) lower and (**b**) higher magnification.

**Figure 2:**
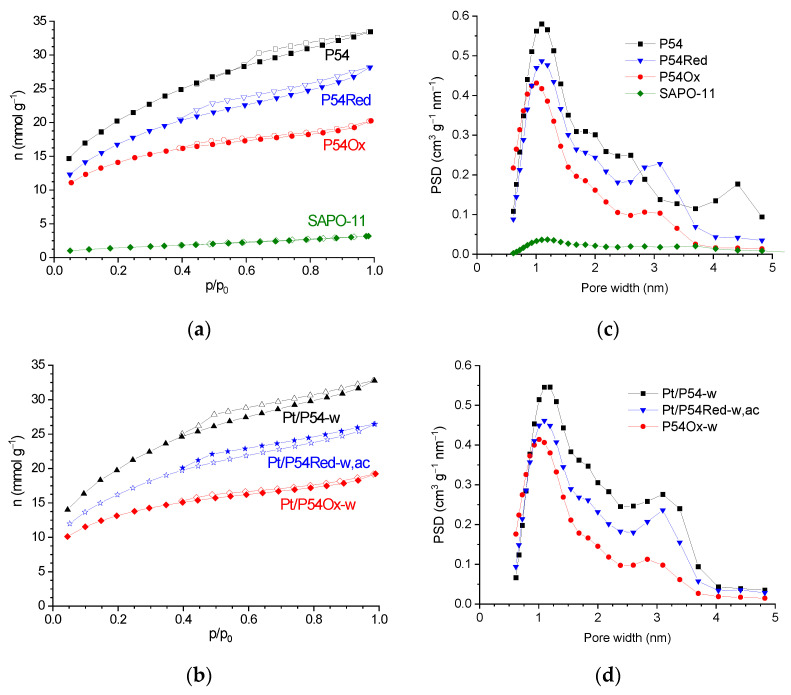
(**a**) N_2_ adsorption–desorption isotherms of the prepared ACs and a commercial SAPO-11 zeolite, as well as of (**b**) selected prepared catalysts (closed and open symbols correspond to the adsorption and desorption branches, respectively). Subfigures (**c**,**d**) show the respective PSD curves.

HR-TEM (high-resolution transmission electron microscopy) micrographs (Figure 1) reveal that the unmodified AC P54 displays a rough surface and extensive porosity. This porosity was probed via N_2_ adsorption–desorption experiments at −196 °C. The resulting isotherm (Figure 2a) can be considered a hybrid of types I (b) and IV, according to IUPAC (International Union of Pure and Applied Chemistry) classification [43], typical of microporous and mesoporous solids, respectively. These findings are confirmed by the corresponding pore size distribution (PSD) curves (Figure 2c) and textural properties (Table 1), with the V_mic_ (volumes of micropores) and V_mes_ (volume of mesopores) found to be equal to 0.72 and 0.55 cm^3^ g^−1^, respectively. This porosity resulted in a high specific surface area of 1643 m^2^ g^−1^.

The XPS (X-ray photoelectron spectroscopy) analysis of P54 revealed the presence of carbon, oxygen, phosphorus, and silicon in proportions of 85.9, 11.9, 1.9, and 0.4 wt%, respectively (Figure S1a in the SM (Supplementary Materials); Table 2). Silicon remained from the composition of the original biomass, while phosphorus resulted from the activation with H_3_PO_4_.

The HR-XPS C 1*s* core level spectrum of P54 (Figure S2a in the SM) was decomposed into four contributions assigned mainly to unfunctionalized and adventitious carbons (CI peak, 284.8 eV); C–O (CII, 286.2 eV); C=O (CIII, 287.4 eV); COO^−^ (CIV, 288.8 eV) [44]. In turn, the HR-XPS O 1*s* core level spectrum was decomposed into two peaks (Figure S2d in the SM) assigned mainly to oxygen double-bounded (OI, 531.7 eV) and single-bounded to carbon (OII, 533.4 eV) (see pertinent discussion in Reference [24]). The relative contribution of each peak is reported in Table 3.

**Figure 3:**
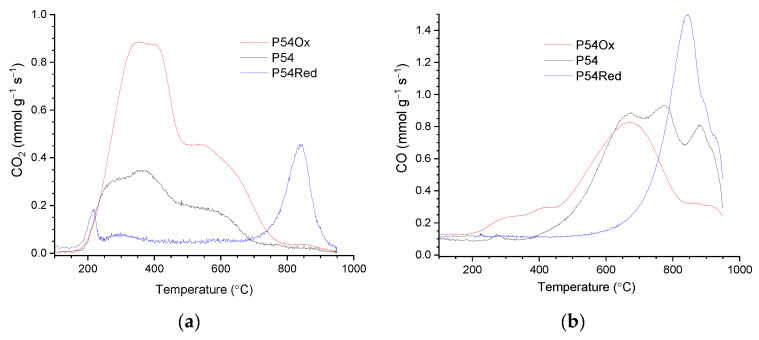
(**a**) CO_2_ and (**b**) CO-TPD profiles of the prepared ACs.

**Figure 4:**
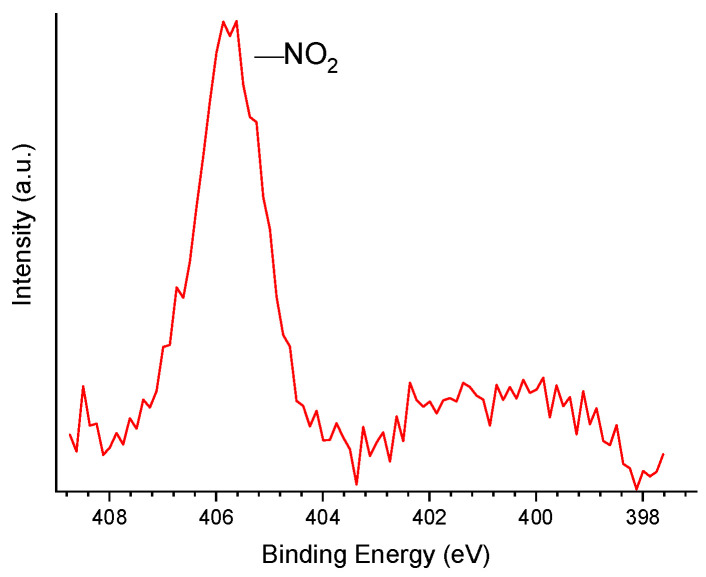
HR-XPS N 1*s* core level spectrum of P54Ox.

P54 showed intense CO_2_ and CO emissions during TPD/MS (temperature-programmed desorption/mass spectrometry) analysis (Figure 3a and Figure 3b, respectively), which was consistent with its relatively high O content (11.9 wt%, Table 2). The deconvolution of the CO_2_ and CO-TPD profiles of P54 was presented in a previous work (Figure 4 in reference [45]). The CO_2_ profile was decomposed into five contributions centered at 260, 363, 500, 606, and 794 °C. The first three were assigned to stronger carboxylic acids, weaker carboxylic acids, and anhydrides, respectively, whereas the other two were attributed to lactones. The CO-TPD profile was also decomposed into five contributions, which were assigned to anhydrides, which release CO_2_ and CO simultaneously (500 °C); phenol/ether (650 and 780 °C); and ketones/quinones (886 and 930 °C).

The titration data (Table 4) revealed that P54 possesses a high content of both strong (0.55 mmol g^−1^) and weak (1.21 mmol g^−1^) acidic groups, alongside a low content of medium acidic groups (0.07 mmol g^−1^). These results are consistent with the TPD/MS analysis given that, as depicted elsewhere [24], it has been assumed that strong acids comprise carboxylic acids and anhydrides, while weak and medium-strength acids correspond mainly to phenols and lactones, respectively. In turn, the content of the basic groups was zero.

The chemical compositions of the prepared ACs were also probed by elemental analysis (EA). For P54, the C, H, and N contents were 84.2, 1.4, and 0.3 wt%, respectively, with a H/C atomic ratio of 0.20 (Table 4).

#### 2.1.2. Thermal Treatment of P54 in a H_2_ Atmosphere

In a previous work [24], a commercial AC sample was heated to 800 °C in an inert atmosphere (N_2_) aiming to remove the functional groups. However, a certain number of these groups persisted, even in the treated material. The explanation for this behavior is that carbon atoms with incomplete valences and unpaired electrons remain after heat treatment in an inert atmosphere, which can subsequently react with atmospheric oxygen and water vapor to generate new acidic oxygenated groups [46,47]. Therefore, in this work, P54 was heat-treated in a H_2_ reductive atmosphere in an attempt to stabilize the reactive carbon atoms.

The reductive treatment up to 800 °C resulted in considerable weight loss (12.4 wt%) and decreases in porosity and specific surface area (V_mic_, V_mes_, and SSA were 0.60 cm^3^ g^−1^, 0.32 cm^3^ g^−1^, and 1367 m^2^ g^−1^ for the treated AC P54Red, respectively, while the corresponding values for the unmodified AC P54 were 0.72 cm^3^ g^−1^, 0.55 cm^3^ g^−1^, and 1643 m^2^ g^−1^, respectively (Table 1)). The weight loss can be mainly attributed to the release of CO_2_ and CO resulting from the decomposition of the oxygenated acidic groups, similar to what was observed during the TPD analysis of P54 (Figure 3). Furthermore, the release of H_2_O, H_2_, and CH_4_ provides minor contributions, as reported elsewhere [24]. In turn, the reduction in porosity and specific surface area can be related to the aromatization process that takes place during thermal treatment above ~600 °C, leading to material shrinkage and consequent pore contraction [24,25,26]. This aromatization can be confirmed by the decrease in the H/C ratio (from 0.20 to 0.08, Table 4).

Concerning the TPD profiles of P54Red, they presented only slight CO_2_ and CO emissions until the temperature approached 800 °C (Figure 3). In contrast, relatively intense CO and CO_2_ peaks with roughly similar profiles were observed at higher temperatures, with a maximum at around 840 °C. The CO peak is assigned to the carbonyl groups, while the less intense CO_2_ peak is assumed to result mainly from a partial oxidation of CO on the AC surface, as previously discussed in Reference [24]. In accordance with the TPD profiles, the HR-XPS C 1*s* peak corresponding to the carboxylic groups (CIV peak) disappeared after the treatment, while the intensity of the peak relative to the carbonylic groups (CIII) slightly increased (Figure S2b in the SM; Table 3).

Consistent with the TPD analyses, the titration data (Table 4) showed that the reductive treatment caused a pronounced reduction in the content of the acidic groups (from 1.83 to 1.15 mmol g^−1^). Simultaneously, there was some increase in the content of the basic groups (from zero to 0.23 mmol g^−1^), which are frequently associated with pyrone-like structures and π-electrons on the basal planes of carbons [24,41]. Due to the pronounced removal of the acidic oxygenated groups, the oxygen content decreased from 11.9 to 8.0 wt% (XPS data, Table 2).

#### 2.1.3. Oxidative Treatment of P54 with HNO_3_

Figure 3a shows that all the CO_2_ emissions during the TPD analyses of the sample treated with HNO_3_ (P54Ox) were more intense than those of the unmodified AC P54, indicating the creation of additional carboxylic acids, anhydrides, and lactones during the treatment. Concerning CO emissions (Figure 3b), they changed little up to around 700 °C (phenol/ether groups), but strongly decreased at higher temperatures. This decrease can be attributed to the oxidative attack of HNO_3_ on the carbonyl groups [24]. Consistent with the TPD analyses, the contents of both strong (mainly carboxylic acids plus anhydrides) and medium-strength acids (mainly lactones) increased (from 0.55 to 1.26 and from 0.07 to 0.42 mmol g^−1^, respectively, Table 4), while the content of weak acids (phenols) showed little change. Furthermore, the HR-XPS C 1*s* core level spectrum showed an increase in the relative intensity of the CIV peak (at ~288.8 eV), assigned to carboxylic carbons (Figure S2c in the SM; Table 3). As a consequence of the insertion of acidic oxygenated groups, the O content (determined by XPS) pronouncedly increased (from 11.9 to 32.4 wt%, Table 2).

The treatment with HNO_3_ also increased the N content, from 0.0/0.3 to 2.4/1.6 wt% (XPS (Table 2)/EA (Table 4)). The HR-XPS N 1*s* core level spectrum of P54Ox (Figure 4) revealed that N was incorporated as nitro groups (–NO_2_), resulting in a peak centered at 405.7 eV (see pertinent discussion in Reference [24]).

The treatment with HNO_3_ reduced the porosity (V_mic_ and V_mes_ decreased from 0.72 and 0.55 cm^3^ g^−1^ to 0.51 and 0.17 cm^3^ g^−1^, respectively, Table 1) and specific surface area (from 1643 to 1129 m^2^ g^−1^, Table 1). This usual behavior can be attributed to two factors: (i) the formation of acidic oxygenated groups capable of blocking the entrance of narrow pores; (ii) the collapse of pore walls due to oxidation [24,48,49].

### 2.2. Searching for Optimal Conditions for Catalyst Preparation

Pt catalysts were prepared using the unmodified (P54) or modified (P54Red or P54Ox) ACs as supports. The preparation involved either incipient wetness impregnation or wet impregnation, with or without the acidification of the impregnating solution with HCl (procedures are detailed in Section 3.3). The obtained catalysts were labeled as follows: the metal symbol (Pt); the AC employed as support is then indicated after a slash; separated by a hyphen, the letter “w” or “i” indicates whether wet or incipient wetting impregnation was used; finally, if the impregnating solution was acidified with HCl, this is indicated by “ac” preceded by a comma. Thus, as an example, Pt/P54Red-w,ac corresponds to the catalyst prepared by the deposition of Pt on the surface of the AC P54Red using wet impregnation with a H_2_PtCl_6_ solution acidified with HCl.

A systematic study involving HDO tests was carried out to determine the optimal conditions for catalyst preparation. Considering that the splitting of the triacylglyceride molecules that constitute the lipid feedstocks results in a complex mixture of fatty acids, this study was carried out using a model compound as the feedstock (lauric acid), which facilitated product characterization and an understanding of the reaction pathways. Subsequently, the catalyst with the highest HDO activity in the tests with lauric acid was employed in the hydroprocessing of a real lipidic feedstock, i.e., coconut oil.

It is worth mentioning that our research group has usually employed coconut oil in HEFA [45,50]. This is because this lipidic feedstock is primarily composed of fatty chains that match the carbon chain length range of jet fuels and, as mentioned in the Introduction, aviation is the sector for which hydrocarbon biofuels offer the most compelling advantages. In addition, since most of the coconut oil fatty chains are C_12_ [51], lauric acid was chosen as the model compound.

The acidic index (AI) of the obtained liquid mixtures served as the key parameter to evaluate the catalysts’ activity for lauric acid HDO. It is important to highlight that the higher the catalyst activity, the higher the lauric acid conversion and therefore, the lower the AI of the obtained product. The AI was also employed to evaluate catalyst activity in coconut oil HDO, which was possible because the triglycerides molecules are first converted into the respective fatty acids [50].

Before presenting the results of the HDO tests, some features concerning catalyst characterization are presented in Section 2.2.1. These features will subsequently be used to rationalize the catalysts’ HDO performance.

#### 2.2.1. Catalyst Characterization

The ICP/OES (inductively coupled plasma/optical emission spectrometry) analyses showed that Pt was effectively deposited on the surface of the AC supports (Table 5). The Pt loading varied from 0.42 to 1.00 wt%, depending on the employed AC and impregnation conditions.

**Figure 5:**
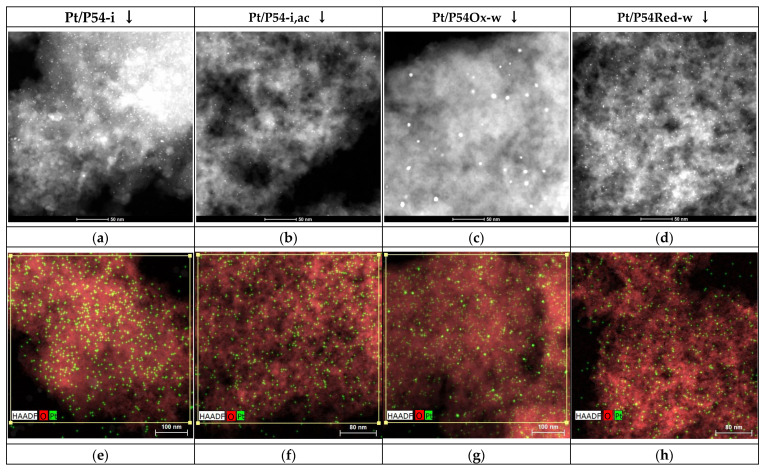

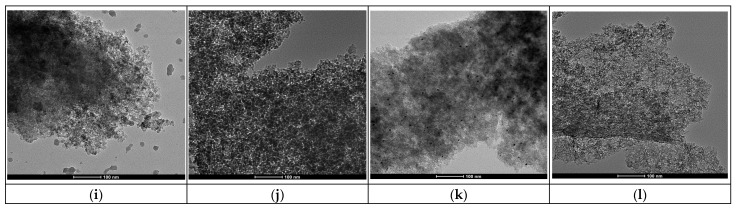
(**a**–**d**) HAADF-STEM, (**e**–**h**) EDX elemental mapping, and (**i**–**l**) HRTEM images of selected catalysts (images corresponding to a given catalyst are in the same column).

HAADF-STEM (Figure 5a–d) and STEM-EDX (energy-dispersive X-ray) elemental mapping images (Figure 5e–h) of selected catalysts reveal small Pt aggregates, a few nanometers in diameter, uniformly distributed over the support surface. The resulting average particle sizes (see the histograms of Pt particle size distribution in Figure S3 of SM) ranged from 2.7 to 6.8 nm (Table 6).

The HR-TEM images in Figure 5i–l show that the rough surface and extensive porosity of the bare ACs were retained in the prepared catalysts. The comparison of the isotherms (Figure 2a,b), PSD curves (Figure 2c,d), and textural properties (Table 1) of the catalysts with those of the respective bare supports confirms that Pt deposition did not significantly reduce the porosity and specific surface area.

Table 2 reports the XPS elemental compositions of the selected catalysts. Notably, the Pt contents determined by XPS were systematically lower than those determined using ICP/OES. Since XPS probes the few uppermost layers of the external surface, while ICP/OES is a bulk technique, these results suggest that most of the Pt was deposited within the pores.

**Figure 6:**
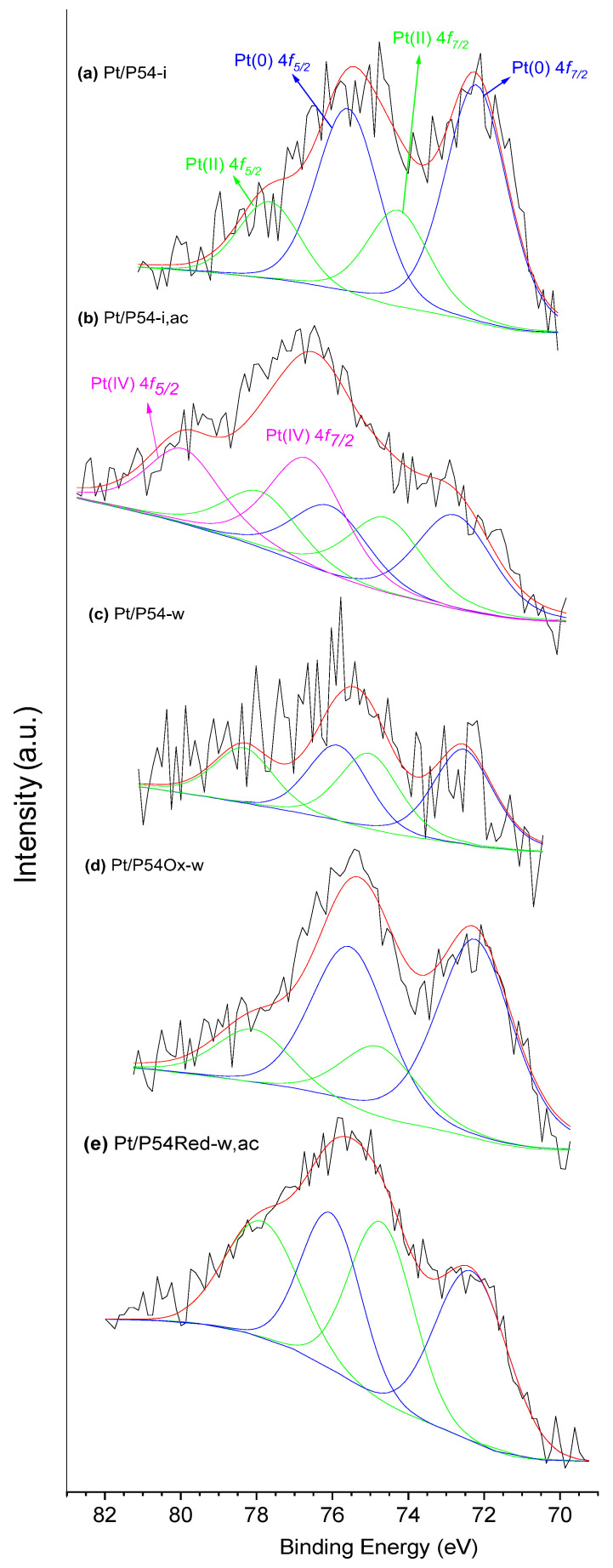
HR Pt 4*f* core level spectra of selected catalysts.

The catalysts’ HR-XPS Pt 4*f* core level spectra displayed a shallow envelope in the range of approximately 70–82 eV (Figure 6). In a general way, the envelopes were deconvoluted into two doublets Pt 4*f*_7/2_-Pt 4*f*_5/2_ [52,53]: one assigned to Pt(0), with the peaks centered in the range of 72.2–72.6 eV and 75.5–76.1 eV, and the other assigned to PtO, with peaks in the range of 74.0–74.8 eV and 77.8–78.3 eV. In the case of Pt/P54-i,ac, a third doublet with peaks at 76.6 and 79.9 eV was verified and assigned to PtO_2_.

A noteworthy observation is that the proportion of Pt in the metallic state (Pt(0)) directly correlated with the average Pt particle size (Table 6). This behavior suggests that, as reported by Singh et al. [52], Pt atoms on the external surface of the particles can be oxidized by air under ambient conditions, leading to a lower proportion of metallic Pt in smaller particles.

**Figure 7:**
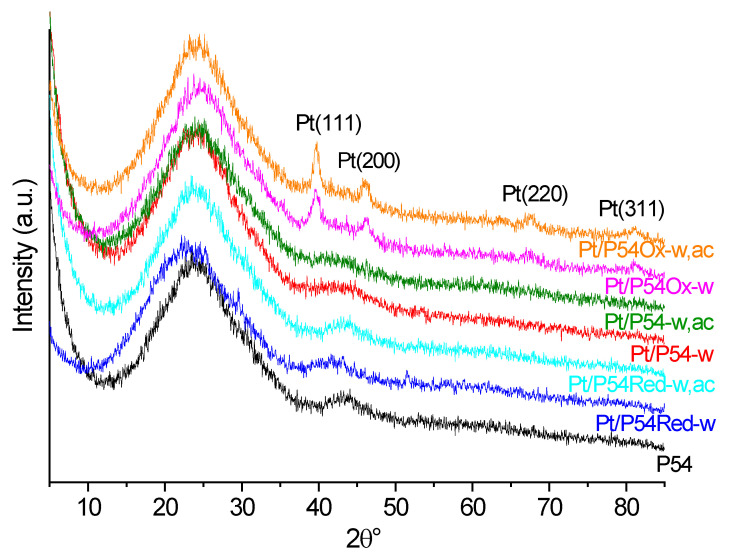
X-ray diffractograms of selected catalysts and the bare AC P54.

Figure 7 displays the XRD (X-ray diffraction) profiles of selected catalysts and for comparison, of the bare AC P54. Except for Pt/P54Ox-w and Pt/P54Ox-w,ac, the diffractograms of the other catalysts exhibit a profile similar to that of P54. They show only two broad peaks due to the (002) and (100) diffraction planes at 2θ at approximately 24 and 43°, which are characteristic of amorphous carbon. On the other hand, the diffractograms of Pt/P54Ox-w and Pt/P54Ox-w,ac present four additional peaks at 36.7, 46.1, 67.5, and 81.1°, assigned to the (111), (200), (220), and (311) diffraction planes of Pt(0), respectively [52]. These results were attributed to the presence of larger Pt particles on the surface of catalysts prepared with the more acidic AC P54Ox as the support. Indeed, the elemental mapping images (Figure 5e–h) show that Pt/P54Ox-w exhibits a lower number of larger Pt particles compared to that of the other catalysts. Accordingly, Pt/P54Ox-w presents the largest average particle size (6.8 nm) reported in Table 6. Remarkably, this value was the only one substantially above the X-ray scattering coherence length of Pt (in the range of around 4.0–4.5 nm, as determined by Schmies et al. [54]). This explains why Pt/P54Ox-w was the only sample to present XRD peaks corresponding to metallic Pt (Figure 7).

#### 2.2.2. Blank Tests

Two 8 h blank tests with lauric acid were carried out, one without any support or catalyst in the reaction system, and another with only the bare AC P54 added (entries 1 and 2 in Table 7). The obtained products presented AIs of 233.8 and 220.8, respectively, which were only slightly lower than the theoretical value calculated for lauric acid (280.0). These results reveal that, at 375 °C, the thermal effect and the bare AC have little influence on the HDO process on their own.

#### 2.2.3. Effects of the Impregnation Methodology

To evaluate the effects of the impregnation methodology on the properties and activity of the resulting catalysts, Pt was deposited onto the surface of the unmodified AC P54 using either incipient wetness impregnation (Pt/P54-i) or wet impregnation (Pt/P54-w), initially without HCl addition to the impregnating solution. Both resulting catalysts presented relatively high HDO activity: the products obtained after 8 h tests for lauric acid HDO using Pt/P54-i and Pt/P54-w exhibited AIs of 13.0 and zero, respectively (entries 3 and 4 in Table 7). The higher activity of the catalyst prepared by wet impregnation can be attributed to its (i) higher Pt content, which was 0.65 wt% for Pt/P54-w and 0.42 for Pt/P54-i (ICP/OES data, Table 5); and (ii) higher Pt dispersion, evidenced by the smaller average Pt particle size of Pt/P54-w (3.9 nm) compared to Pt/P54-i (5.2 nm) (Table 6).

The higher Pt dispersion presented by Pt/P54-w can be explained as follows. During impregnation, it is believed that Pt-containing species are promptly adsorbed on the most accessible support surface, that is to say, on the external surface and the surface of wider pores. Consequently, if incipient wetness impregnation is employed, the material would be dried and calcined with most of the Pt deposited on these surfaces. In this scenario, solute diffusion into the pore network would be the controlling step, resulting in a relatively low metal dispersion. Conversely, with wet impregnation, the prolonged contact between the support surface and the abundant impregnating solution could reverse the initial adsorption on the most accessible surface, allowing the Pt-containing species to penetrate into smaller pores. In this scenario, the process would be under thermodynamic control, and a higher metal dispersion would be attained. This hypothesis is corroborated by the finding that, compared to Pt/P54-i, Pt/P54-w presented a higher Pt content determined by ICP/OES (a bulk technique), but a lower Pt content determined by XPS (a technique that probes the external surface). The Pt contents determined by ICP/OES were 0.65 and 0.42 wt% for Pt/P54-w and Pt/P54-i, respectively (Table 5), while the corresponding values determined by XPS were 0.1 and 0.5 wt%, respectively (Table 2).

In turn, the lower Pt content of Pt/P54-i could be explained by solution spillage during incipient wetness impregnation, where part of the metal is invariably carried with the solution and deposited onto the vessel walls (mainly the bottom wall). On the other hand, if wet impregnation is employed, practically all metal-containing species would be adsorbed during the 24 h stirring step, so that only a small portion of metal would be lost due to deposition on the vessel walls during the drying step.

#### 2.2.4. Effects of the Treatment of the P54 AC with HNO_3_

To evaluate the effects of treating the P54 AC with HNO_3_, the resulting P54Ox support was then impregnated with the H_2_PtCl_6_ solution (first, without HCl addition). Since wet impregnation rendered the best catalysts in previous tests with the unmodified P54 support (Section 2.2.3), this impregnation methodology was applied here and in subsequent experiments.

Despite its higher Pt content (determined by ICP/OES, Table 5), Pt/P54Ox-w exhibited significantly lower HDO activity than that of Pt/P54,w. The AI values for the products obtained in the 5 h tests for lauric acid HDO using Pt/P54Ox-w and Pt/P54-w were 73.8 and 1.2, respectively (entries 6 and 5 in Table 7).

The higher Pt loading on Pt/P54Ox-w (compared to Pt/P54-w) is consistent with the proposal by van Dam and van Bekkum [55] that lone electron pairs on oxygen atoms and π-electrons from carbon basal planes act as active sites for anchoring Pt-containing complexes, coordinating to the metal. In this sense, the higher Pt loading on Pt/P54Ox-w would be explained by the much higher content of acidic oxygenated functional groups on the P54Ox support.

In turn, the lower activity of Pt/P54Ox-w can be attributed to its lower Pt dispersion, which was already diagnosed by the presence of larger Pt particles (see Section 2.2.1). This lower Pt dispersion can be understood by considering the abundance of carboxylic acids over P54Ox. As shown in Section 2.1.1, carboxylic acids decompose below ~400 °C, which means that they decompose during the reduction step of catalyst preparation. Consequently, as suggested by de Miguel et al. [53] and Coloma et al. [56], Pt-species anchored to these groups would acquire mobility, thus favoring metal sintering.

One could attribute the lower Pt dispersion over Pt/P54Ox-w,ac to the high concentration of negatively charged groups on the support surface (resulting from the deprotonation of abundant acid groups), which would promote electrostatic repulsion with the Pt-containing anions, thus favoring Pt sintering. However, this phenomenon would likely occur in basic media, as reported by Okhlopkova [41], but not at the acidic pH of a H_2_PtCl_6_ solution, where the deprotonation of acidic surface groups is unfavorable.

#### 2.2.5. Effects of Thermally Treating the P54 AC in a H_2_ Atmosphere

To evaluate the effects of thermally treating the P54 AC in a H_2_ atmosphere, the resulting P54Red support was then impregnated with the H_2_PtCl_6_ solution using the wet impregnation methodology (first, without HCl addition). The obtained catalyst (Pt/P54Red-w) exhibited a slightly lower HDO activity than that of its counterpart prepared with the unmodified support (Pt/P54-w). After a 5 h test for lauric acid HDO, Pt/P54Red-w and Pt/P54-w yielded products with AIs of 2.6 and 1.2, respectively (entries 7 and 5 in Table 7).

The high HDO activity displayed by Pt/P54Red-w, despite its significantly lower content of oxygenated functional groups on P54Red compared to the unmodified AC P54 (see Section 2.1.2), provides evidence that basic sites such as carbonyls and π-electrons are active for anchoring Pt-containing species. Furthermore, as initially proposed by Van Dam and van Bekkum [55] and later confirmed by Sepúlveda-Escribano et al. [23] and de Miguel et al. [57], oxygen-poor surfaces (like P54Red) are prone to be oxidized by [PtCl_6_]^2−^ ions (Equation (1)), and the resulting phenolic groups would also contribute to anchoring the formed [PtCl_4_]^2−^ ions.

CH + [PtCl_6_]^2−^ + H_2_O ⇆ [PtCl_4_]^2−^ + COH + 2H^+^ + 2Cl^−^ (1)

Remarkably, the phenol groups did not decompose during the reduction step of catalyst preparation, which would thereby favor Pt dispersion on Pt/P54Red-w. Furthermore, we believe that, for steric reasons, the coordination of the resulting square planar complex [PtCl_4_]^2−^ (Equation (2)) is favored compared to that of the octahedral complex [PtCl_6_]^2−^ (Equation (3)).

S: + [PtCl_4_]^2−^ ⇆ [S—PtCl_3_]^−^ + Cl^−^ (2)

S: + [PtCl_6_]^2−^ ⇆ [S—PtCl_5_]^−^ + Cl^−^ (3)

#### 2.2.6. Effects of Acidifying the Impregnating Solution with HCl

To investigate the effects of acidifying the impregnating solution with HCl, the syntheses of all catalysts were repeated, but with HCl added to the H_2_PtCl_6_ solution. The effects of this operation depended on the employed support and impregnation methodology, as depicted in the sequence.

For catalysts prepared by incipient wetness impregnation, HCl addition to the impregnating solution led to a catalyst with increased HDO activity. The 8 h test for lauric acid HDO with the catalyst prepared with HCl addition (Pt/P54-i,ac) yielded a product with an AI of 1.9 (entry 8 in Table 7), significantly lower than the AI of 13.0 (entry 3 in Table 7) verified for the product obtained using its counterpart prepared without HCl addition (Pt/P54-i). These results can be attributed to (i) the higher Pt content of Pt/P54-i,ac (0.58 wt%) compared to that of Pt/P54-i (0.42 wt%), as determined by ICP/OES (Table 5) and (ii) the improved Pt dispersion over Pt/P54-i,ac, evidenced by its smaller average Pt particle size (3.0 nm) compared to that of Pt/P54-i (5.2 nm) (Table 6). A likely explanation for these findings is that, as Cl^−^ is a product of all the reactions in Equations (1)–(3), HCl addition to the impregnating solution would shift the equilibria to the left, thereby disfavoring the coordinative adsorption mechanism. Therefore, we believe that the adsorption of Pt-containing species is slowed down, allowing them to penetrate deeper into the pore network, which in turn leads to a higher Pt dispersion. Furthermore, this scenario would end up reducing the Pt lost to the vessel’s walls, which would justify the higher Pt loading measured by ICP/OES for Pt/54-i,ac compared to that for Pt/54-i (Table 5).

Conversely, for catalysts prepared by wet impregnation, HCl addition to the impregnating solution proved detrimental to HDO activity when using the unmodified AC P54 or the oxidized AC P54Ox as the support. The 5 h tests for lauric acid HDO using Pt/P54-w,ac and Pt/P54-w yielded products with AI 7.0 and 1.2, respectively (entries 10 and 5 in Table 7), whereas, for Pt/P54Ox-w,ac and Pt/P54Ox-w, the corresponding values were 81.0 and 73.6 (entries 11 and 6 in Table 7). These results can be interpreted as follows. On one hand, when using incipient wetness impregnation, the addition of HCl is important to disfavor the rapid adsorption of Pt-containing species on the most accessible surface, thereby allowing deeper penetration of Pt into the pore network; on the other hand, wet impregnation provides appropriate diffusion of Pt-containing species on its own, as depicted in Section 2.2.3. Therefore, for P54 and P54Ox ACs, the sole effect of adding HCl to the impregnating solution is to disfavor the coordination of Pt-containing species to the support surface (see pertinent considerations in the previous paragraph). Consequently, Pt sintering is favored during the subsequent reduction step, resulting in lower Pt dispersion.

Unlike what was observed when using the more acidic ACs P54 and P54Ox as supports, in the case of the reduced AC P54Red, the addition of HCl to the impregnating solution resulted in a catalyst with improved HDO activity. The AIs of the products obtained in the 5 h tests for lauric acid HDO using Pt/P54Red-w,ac and Pt/P54Red-w were 0.4 and 2.6, respectively (entries 12 and 7 in Table 7). The higher activity presented by Pt/P54Red-w,ac can be attributed to its greater Pt dispersion, which is evidenced by the large number of small Pt particles observed in the EDX elemental mapping image of this catalyst (Figure 1h). Consistent with the visual analysis, Pt/P54Red-w,ac was the catalyst that exhibited the smallest average Pt particle size (2.7 nm, Table 6). We believe that the improved Pt dispersion achieved by the addition of HCl to the impregnating solution when using the more basic AC P54Red as the support can be explained as follows: the low pH of the impregnating solution would favor the protonation of the basic sites, resulting in a positively charged surface. This would then promote strong attractive electrostatic interaction with the Pt-containing anions, thereby disfavoring Pt sintering and increasing Pt dispersion.

At this point, it is worth highlighting that Regalbuto’s research group has published several works considering electrostatic interaction as the main mechanism for the adsorption of Pt-containing species (see, for example, References [58,59]). However, we believe this is true only in the specific cases of (i) basic ACs impregnated with anionic Pt-containing species (e.g., PtCl_6_^2−^) in acidic pH and (ii) acidic ACs being impregnated with cationic Pt-containing species (e.g., Pt(NH_3_)_4_^2+^) in basic pH. In the first case (which corresponds to the preparation of Pt/P54Red-w,ac in the present work), the basic sites would be protonated, and the positively charged surface would thereby promote the electrostatic adsorption of the Pt-containing anions. In the second case, the acidic sites would be deprotonated, and the negatively charged surface would thereby promote the electrostatic adsorption of Pt-containing cations. In other scenarios, we believe that the coordinative mechanism portrayed in Equations (1)–(3) plays the main role in the adsorption of Pt-containing species. Otherwise, it would not be possible to explain why, in the present work, the acidification of the impregnating solution led to a decrease in catalyst activity when using the more acidic supports P54 and P54Ox.

#### 2.2.7. Effect of In Situ Re-Reduction

Given the considerable content of oxidized Pt observed in the prepared catalysts, even after the reduction step (due to the reaction with atmospheric air, as depicted in Section 2.2.1), we initially performed an in situ catalyst re-reduction step before the HDO tests, as detailed in Section 3.5. However, we later decided to evaluate whether this re-reduction step was truly pertinent. For that, the 5 h test for lauric acid HDO using the Pt/P54Red-w,ac catalyst was repeated, but without the re-reduction step. Somewhat surprisingly, the results revealed that the re-reduction step is not only unnecessary, but it even slightly reduces the catalysts’ HDO activity. Suppressing the re-reduction step yielded a product with an AI of zero, whereas the value observed for the product from the equivalent test with the re-reduction step was 0.4 (entries 13 and 12 in Table 7, respectively). These results prove that the H_2_ atmosphere and the relatively high temperature (375 °C) employed during the HDO tests are sufficient to reduce the oxidized Pt formed on the surface of the Pt nanoparticles due to the exposure to the atmospheric air, whereas the introduction of a re-reduction step at 400 °C is likely to provoke some Pt sintering, thereby decreasing the catalysts’ activity.

Since the re-reduction step was found to be detrimental to the catalysts’ activity, it was omitted in the subsequent HDO tests with coconut oil.

### 2.3. Composition of the Product of Lauric Acid HDO

**Figure 8:**
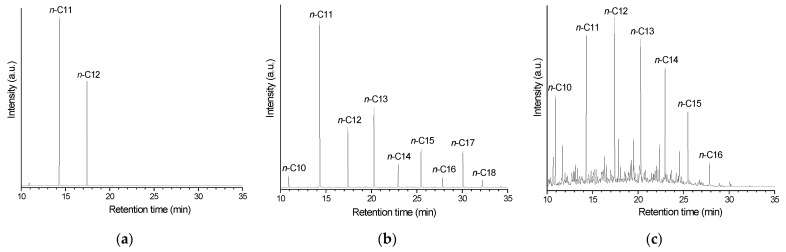
GC/FID chromatograms of the product obtained after the tests for (**a**) lauric acid (5 h) and (**b**) coconut oil (7 h) HDO using the catalyst Pt/P54Red-w,ac (without in situ re-reduction); (**c**) the commercial petroleum-based jet fuel QAV-1.

Figure 8a shows the GC/FID (gas chromatography/flame ionization detector) chromatogram of the product obtained after the 5 h test for lauric acid HDO using the Pt/P54Red-w,ac catalyst. This chromatogram presents only two prominent peaks, assigned to *n*-undecane (*n*-C_11_) and *n*-dodecane (*n*-C_12_). The former resulted from decarboxylation (DCX) and decarbonylation (DCN) reactions (Equations (4) and (5)), while the latter was formed via hydrogenation/dehydration reactions (Equation (6)). The relative areas for the *n*-C_11_ and *n*-C_12_ peaks were 64.4 and 35.6%, respectively.



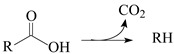

(4)




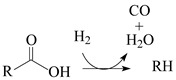

(5)




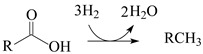

(6)


The absence of peaks corresponding to shorter and branched chains indicates that cracking and isomerization reactions were negligible, in accordance with the relatively low acidity of the ACs (as reported elsewhere [50], the acidity of the support is known to play an essential role in hydrocracking and hydroisomerization reactions).

### 2.4. HDO Tests with Coconut Oil

As Pt/P54Red-w,ac was the catalyst that presented the highest activity for lauric acid HDO, it was selected for the subsequent tests with coconut oil. Unlike in the tests with lauric acid, the product obtained in the 5 h test with coconut oil exhibited a residual acidity, resulting in an AI of 1.4 (entry 14 in Table 7). This result is attributed to the fact that the triacylglyceride molecules in the oil must first be cleaved into propane and the corresponding fatty chains before the fatty chains are deoxygenated. As expected, this additional reaction step involving bulky molecules (as is the case with triacylglycerides) reduces the rate of conversion to alkanes. To overcome this issue, the reaction time for coconut oil HDO was increased to 7 h, which resulted in a product with an AI of zero (entry 15 in Table 7). Accordingly, the chromatogram of the obtained product (Figure 8b) presented only peaks corresponding to alkanes (basically *n*-alkanes), which covered a large chain length range, with emphasis on *n*-C_11_ and *n*-C_12_. These results are consistent with the coconut oil composition [51] and the HDO pathways presented in Equations (4)–(6).

As can be seen, the intensity of the peaks corresponding to alkanes with an odd number of carbons is consistently higher than that of the peaks related to alkanes with an even number of carbons. This finding is consistent with the predominance of the DCX and DCN pathways over the hydrogenation/dehydration pathway, as verified in the tests with lauric acid (Section 2.3).

As expected (see pertinent discussion in the beginning of Section 2.2), the alkanes present in the product of coconut oil HDO are in the same chain length range as those found in the petroleum-based commercial jet fuel QAV-1 (supplied by Petrobras Oil Company, Rio de Janeiro, RJ, Brazil; compare Figure 8b,c). However, it is worth mentioning that, unlike the product of coconut oil HDO, the chromatogram of QAV-1 exhibits numerous peaks beyond those corresponding to linear alkanes (i.e., branched isomers, naphthenics, and aromatics).

While it would be desirable to compare the HDO activity of the catalysts prepared in this work with that of other typical HDO catalysts, direct comparison with studies reported in the literature is rather difficult due to the wide range of reaction systems, feedstocks, and reaction conditions employed. For example, as noted in the Introduction, the two existing studies concerning the use of AC-supported Pt catalysts in the HDO of lipid feedstocks (References [38,39]) were carried out in continuous systems, which precludes a comparison with the results obtained in batch mode in the present work.

Nevertheless, we have previously reported [45] results concerning the use of two sulfided Mo-based catalysts in HDO tests using the same feedstock and reaction system as in the present work, under relatively similar reaction conditions. For example, the same catalyst/feedstock mass ratio of 0.05 was used, although at a somewhat lower reaction temperature. Both catalysts contained Ni as a promoter. One of them (sulf-Ni,Mo/P54-w,4) was prepared by our team using the unmodified AC P54 as the support. The other (NiMoS/Al_2_O_3_) was a commercial catalyst supplied by Petrobras Oil Company (Rio de Janeiro, RJ, Brazil) and was supported on γ-Al_2_O_3_. After 3 h of coconut oil HDO at 340 °C, sulf-Ni,Mo/P54-w,4 and NiMoS/Al_2_O_3_ rendered products with AI values of 1.1 and 6.3, respectively (entries 17 and 18 in Table 7). In turn, after 3 h, the catalyst Pt/P54Red-w,ac (prepared in the present work) yielded a product with a considerably higher AI of 15.4, even though a higher reaction temperature (375 °C) was employed (entry 16 in Table 7).

These results can lead to the inference that Pt is less efficient than sulfided Mo in HDO. However, it is important to note that Pt/P54Red-w,ac contained less than 1 wt% of Pt, whereas sulf-Ni,Mo/P54-w,4 and NiMoS/Al_2_O_3_ yielded almost 20 wt% of Mo, besides the Ni added as a promoter (the characterizations of sulf-Ni,Mo/P54-w,4 and NiMoS/Al_2_O_3_ are reported in References [45] and [50], respectively). At this point, it is worth highlighting that it is usual to employ a much higher metal content in Mo-based than in Pt catalysts (see, just as a few examples, References [8,50,60,61] and [4,30,31,37,38] for sulfided Mo and Pt catalysts, respectively). The main reason is the high cost of Pt, which makes it essential to optimize the catalytic activity per metal mass unit. This is achieved by loading low amounts of Pt per unit of support surface area, thereby improving metal dispersion.

We also tested another Pt catalyst prepared by our team using a commercial SAPO-11 zeolite as the support. This catalyst was previously characterized and employed to promote the hydroisomerization of *n*-alkanes [50]. However, this catalyst showed quite low HDO activity, with the product obtained in a 5 h test for coconut oil HDO presenting an AI of 153.8 (entry 19 in Table 7). We suspect that the main reason for the low HDO activity presented by Pt/SAPO-11 is related to the low available porosity of the SAPO-11 support. To allow comparison with the prepared ACs, the N_2_ adsorption/desorption isotherms and respective PSD curve of SAPO-11 are presented in Figure 2a,c. Further investigation would be required to draw more definitive conclusions about this matter; however, this is beyond the scope of the present work.

### 2.5. Catalyst Reusability

**Figure 9:**
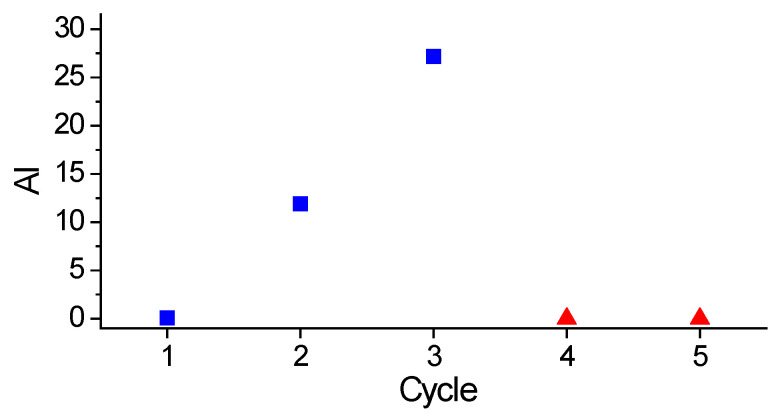
AI values of the products obtained throughout successive cycles of coconut oil HDO; the first three cycles (blue squares) were conducted without in situ catalyst re-reduction, whereas the fourth and fifth cycles (red triangles) were carried out with in situ catalyst re-reduction before each new cycle.

The reusability of Pt/P54Red-w,ac was investigated by reusing the same catalyst sample in consecutive cycles of coconut oil HDO. After each cycle, the spent catalyst was separated from the liquid phase by filtration and then dried overnight at 100 °C. Small catalyst losses (about 2–4%) were compensated by adding equivalent amounts of fresh catalyst. Figure 9 shows that, without performing in situ re-reduction, the catalyst experienced a significant and continuous loss of activity over successive reaction cycles. The AI values of the products obtained after the first, second, and third cycles were zero, 11.9, and 27.2, respectively. Conversely, when an in situ re-reduction step was carried out before a new cycle (as in the fourth and fifth cycles), the catalyst activity was restored. These results indicate that, during each cycle, the oxygenated reaction products (i.e., CO_2_, and H_2_O) cause Pt oxidation. Remarkably, this oxidation, which occurred under relatively severe temperature and pressure conditions, appears to be deeper than the shallow oxidation that occurs due to the simple exposure of the catalyst to atmospheric air (see pertinent discussion in Section 2.2.7), making the reaction conditions insufficient to quickly restore the catalyst activity at the beginning of a new reaction cycle. Therefore, an in situ re-reduction step was necessary to restore the catalyst activity to a level comparable to that of the fresh catalyst.

It is noteworthy that the loss of HDO activity was much higher for the Pt catalysts than for the sulfided Mo-based catalyst referred to in Section 2.4. While the AIs of the products obtained in the present work after the second and third HDO cycles with the Pt/P54Red-w,ac catalyst were 11.9 and 27.2, respectively, the corresponding values previously reported for the products obtained with the Ni,Mo/P54-w,4 catalyst were only 0.2 and 1.1, respectively [45]. However, if on the one hand, the activity loss was higher for the Pt catalyst, on the other hand, recovering the activity of sulfided Mo-based catalysts is more challenging. This difficulty stems from the need for sulfiding agents, which, as mentioned in the Introduction, exhibit a risk of product contamination with sulfur. Finally, it should be emphasized that these results were obtained in batch mode; therefore, further studies should be conducted to assess catalyst stability under continuous reaction conditions.

## 3. Materials and Methods

### 3.1. P54 Preparation

A dried endocarp of coconut shell (*Cocos nucifera* L.) originating from the Federal District region of Brazil, was used as raw material for synthesis of the AC P54. The endocarp was crushed and sieved, and the fraction in the range of 40–80 mesh (0.177–0.400 mm) was chemically activated with H_3_PO_4_ (85%; Vetec, Speyer, Germany), following a procedure described elsewhere [40]. Briefly, the raw material was impregnated with an aqueous solution containing the desired amount of H_3_PO_4_ and heated to 450 °C under an inert atmosphere (N_2_). Afterwards, the carbonized material was washed with deionized water to remove the chemicals.

### 3.2. P54 Modification

P54 was treated with a 1.0 mol L^−1^ HNO_3_ solution. For this purpose, 20 g of P54 were added to a beaker jar containing 200 mL of the acidic solution. The mixture was kept under reflux (~75 °C) and magnetic stirring for 1 h. After cooling, the solid was separated by filtration, washed with deionized water until a stable pH of approximately 6 was reached, and then dried at 110 °C overnight.

P54 was also treated up to 800 °C (2 h; 5 °C min^−1^) under a H_2_ atmosphere (~100 mL min^−1^) in a horizontal tubular furnace.

### 3.3. Preparation of the Pt Catalysts

Pt was deposited on the ACs from an aqueous solution of hexachloroplatinic acid (H_2_PtCl_6_ · 6H_2_O; ≥99.9%; Sigma Aldrich, St. Louis, MI, USA) via two different methodologies: incipient wetness impregnation and wet impregnation. In the former, the impregnating solution was added dropwise over the AC, so that it diffused through the pores by capillarity. The solution volume (determined through preliminary tests on aliquot samples) corresponded to the maximum volume that could be added before external wetness was observed on the material surface. Afterwards, the material was dried overnight at 110 °C. In the case of wet impregnation, an excess of impregnating solution was used (around 13 mL per gram of AC). The system was closed and kept under stirring for 48 h at 50 °C. After that, the excess water was evaporated by opening the vessel. Finally, the material was dried overnight at 110 °C.

The employed impregnating solutions contained H_2_PtCl_6_ in a concentration corresponding to 1.0 wt% Pt relative to the AC support. In some tests, the solution was acidified with HCl in an amount equivalent to eight times the molar amount of Pt.

After impregnation, the material was reduced under a H_2_ atmosphere (~100 mL min^−1^) at 400 °C (2 h).

### 3.4. Characterization of ACs and Catalysts

The textural properties were characterized by adsorption/desorption isotherms of N_2_ (−196 °C) obtained on a Quantachrome NovaWin 2200e volumetric automatic system (Boynton Beach, FL, USA). Specific surface area and V_mic_ were determined by applying the Brunauer–Emmett–Teller (BET) and Dubinin–Radushkevich (DR) equations, respectively. The volume of liquid N_2_ adsorbed at p/p_0_ 0.95 was termed V_0.95_, which was considered the sum of V_mic_ and V_mes_. Therefore, V_mes_ was determined by subtracting V_mic_ from V_0.95_. The PSD curves were obtained using AsiQwin software (version 4.0, Quantachrome Instruments, Boynton Beach, FL, USA), applying the quenched solid density functional theory (QSDFT) to the N_2_ adsorption data.

The C, H, and N content of the ACs was determined by EA using a Parkin Elmer analyzer model EA 2400 Series II equipped with an AD6 ultra-microbalance (Waltham, MA, USA). The ash content was determined on a dry basis by heating the material at 600 °C for 6 h in a muffle furnace open to ambient air. The bulk content of Pt was determined by ICP/OES analyses carried out on an Agilent 5100 device (Santa Clara, CA, USA).

TPD/MS analyses were carried out on an automated reaction system model AMI-90R coupled to a Dymaxion quadrupole mass spectrometer (Altamira Instruments, Pittsburgh, PA, USA). The sample (~100 mg) was placed in a U-tube of quartz under Ar flow (10 cm^3^ min^−1^; atmospheric pressure). The system was first heated to 110 °C (10 °C min^−1^; 30 min) to release the physically adsorbed molecules. The temperature was then raised to 950 °C (10 °C min^−1^). The emitted gases were monitored with the mass spectrometer. The CO and CO_2_-profiles were fitted assuming a Gaussian distribution for each peak. The peak areas were correlated to the amount of gas present through a routine daily calibration that consisted of the injection of a known volume of pure gas using a calibrated loop [62].

XPS measurements were performed on a Physical Electronics 5700 spectrometer using the Mg-Kα line (1253.6 eV) generated by a PHI model 04-548 Dual Anode X-rays Source (Chanhassen, MN, USA) operating at 300 W (10 keV and 30 mA). The pressure inside the vacuum chamber was 5 × 10^−7^ Pa. A Perkin Elmer 10-360 hemispherical analyzer (Eden Prairie, MN, USA) with a multi-channel detector was employed. The samples were analyzed at an incidence angle of 45° relative to the surface plane. The BEs were referred to the C 1*s* line of adventitious carbon at 284.8 eV and determined with the resolution of ±0.1 eV. The spectra were fitted assuming a Gaussian–Lorentzian distribution for each peak.

HR-TEM was carried out with a TALOS F200x instrument operating in STEM (scanning transmission electron microscopy) mode at 200 kV and 200 nA, equipped with a HAADF (high-angle annular dark-field) detector (Thermo Fisher Scientific, Waltham, MA, USA). The elemental mapping was carried out on an EDX Super-X system equipped with four X-ray detectors and an X-FEG (field emission gun) beam (Thermo Fisher Scientific, Waltham, MA, USA). Pt particle size distribution was calculated using the free software Image J 1.51k. At least 700 platinum particles from up to three different regions of each catalyst were analyzed to obtain a representative statistical analysis.

XRD diffractograms were obtained on a Rigaku instrument model Miniflex 300 (Akishima-shi, Tokyo, Japan) using Ni-filtered Cu-Kα radiation (λ = 0.15406 nm).

The contents of the acidic and basic groups in the ACs were determined through a procedure adapted from the Boehm titration methodology, as detailed elsewhere [24].

### 3.5. HDO Tests

Lauric acid (99.0%, Sigma Aldrich) and extra virgin coconut oil (98%; Dr. Orgânico, Niterói, RJ, Brazil) were used without further purification as feedstocks in the HDO tests. The tests were carried out in a cylindrical stainless steel reactor with an internal volume of ~100 cm^3^ (a scheme of the reactor is presented elsewhere [50]). For in situ re-reduction, 0.500 g of catalyst was charged into the reactor, which was then purged and pressurized with H_2_ at 20 bars and heated to 400 °C for one hour. After that, the system was cooled down to room temperature, and 10.0 g of feedstock was charged into the reactor under a N_2_ flow. The reactor was purged and pressurized again with H_2_ at 20 bars and heated to 375 °C. The heating took around 45 min, and the reaction time zero was considered to be the moment when the temperature reached 375 °C. After the desired HDO reaction time, the system was cooled down to room temperature, and the liquid product was dried over Na_2_SO_4_ and separated by centrifugation. For the experiments in which re-reduction was not performed, the first heating cycle was omitted.

### 3.6. Characterization of Reaction Products

The liquid reaction products were qualitatively analyzed by GC/MS (gas chromatography/mass spectrometry) using a GC-17a chromatograph interfaced with a QP5050A spectrometer. For quantitative analyses, the GC/FID chromatograms were recorded on a GC-2010 device (Kyoto, Japan). A Rtx-5MS polydimethylsiloxane column (30 m, 25 mm) was used in both GC/MS and GC/FID analyses. All chromatographs, spectrometers, and columns were manufactured by Shimadzu (Kyoto, Japan).

The AI of the reaction products was determined according to the AOCS methodology Cd-3d-63-O.

## 4. Conclusions

A mesopore-rich AC was produced from chemical activation of a dried endocarp of coconut shell with H_3_PO_4_. This AC was modified by two treatments: (i) heating to 800 °C in a reductive H_2_ atmosphere, which removed the acidic oxygenated functional groups, and (ii) treatment in a refluxing HNO_3_ solution, which increased the content of the acidic oxygenated functional groups.

The unmodified and modified ACs were employed as supports in the preparation of Pt catalysts aimed at the synthesis of hydrocarbon biofuels via the hydroprocessing of lipid feedstocks. A systematic study on the effects of the preparation conditions on the properties and performance of the obtained catalysts has been carried out for the first time.

Compared to incipient wetness impregnation, wet impregnation enabled better diffusion of the Pt-containing species through the pore network of the supports, thus leading to higher Pt dispersion and consequently, more active catalysts.

The oxygenated functional groups and π-electrons on the carbon basal planes acted as active sites for the coordination of Pt-containing species. However, the presence of carboxylic acids is unfavorable, as these groups decompose during the Pt reduction step, allowing the Pt to become mobile and to sinter. For this reason, catalysts prepared using the HNO_3_-pretreated AC as the support exhibited low Pt dispersion and consequently, poor HDO activity.

Acidification of the impregnating solution with HCl led to an alternative adsorption mechanism when a more basic AC was used as the support. The low pH of the impregnating solution favored the protonation of the basic sites, resulting in a positively charged surface that promoted strong attractive electrostatic interaction with the Pt-containing anions, thus inhibiting Pt sintering and increasing Pt dispersion.

In this context, the catalyst with the highest HDO activity was the one prepared using the AC subjected to thermal pre-treatment in a H_2_ atmosphere as the support, with Pt deposited by wet impregnation of a H_2_PtCl_6_ solution acidified with HCl. The resulting catalyst promoted a complete deoxygenation of lauric acid and coconut oil, yielding products composed mainly of *n*-alkanes. Catalyst stability was achieved by maintaining Pt in its reduced form throughout successive HDO cycles.

The obtained results showed that AC-supported Pt catalysts show great potential to be used in the production of hydrocarbon biofuels through the hydroprocessing of lipid feedstocks.

## Figures and Tables

**Table 1:** Textural properties of the bare ACs and prepared catalysts.

Sample	^1^ SSA (m^2^ g^−1^)	V_mic_ (cm^3^ g^−1^)	V_mes_ (cm^3^ g^−1^)	V_0.95_ (cm^3^ g^−1^)
P54	1643	0.72	0.55	1.17
P54red	1367	0.60	0.32	0.92
P54Ox	1129	0.51	0.17	0.68
Pt/P54-i	1604	0.60	0.55	1.15
Pt/P54-w	1616	0.60	0.54	1.14
Pt/P54Ox-w	994	0.40	0.26	0.67
Pt/P54Red-w	1314	0.49	0.47	0.96
Pt/P54-i,ac	1646	0.62	0.56	1.19
Pt/P54-w,ac	1591	0.61	0.53	1.14
Pt/P54Ox-w,ac	935	0.39	0.22	0.61
Pt/P54Red-w,ac	1280	0.48	0.43	0.92

^1^ Specific surface area.

**Table 2:** Surface elemental chemical composition of the bare unmodified and modified ACs, as well as of selected catalysts prepared in the present work, as determined by XPS.

Sample	Content (wt%)					
	C	O	N	P	Si	Pt
P54	85.9	11.9	-	1.9	0.4	-
P54red	89.3	8.0	0.2	1.8	0.7	-
P54Ox	61.0	32.4	2.4	3.6	0.6	-
Pt/P54-i	89.4	8.8	-	1.4	-	0.5
Pt/P54-i,ac	71.3	19.4	1.3	5.7	1.7	0.7
Pt/P54-w	81.2	13.1	0.5	2.6	2.5	0.1
Pt/P54Ox-w	76.9	16.4	-	2.8	2.4	0.4
Pt/P54Red-w,ac	73.1	16.4	-	1.4	8.3	0.7

**Table 3:** Relative contributions of C 1*s* and O 1*s* peaks in the HR-XPS core level spectra of the prepared ACs (data concerning each element separately and, in brackets, all identified elements).

Sample	Relative Contribution (%)					
	CI	CII	CIII	CIV	OI	OII
P54	85.1 (73.1)	9.0 (7.7)	4.0 (3.5)	1.9 (1.6)	44.8 (5.3)	55.2 (6.6)
P54red	84.9 (75.8)	7.7 (6.9)	7.4 (6.6)	-	32.5 (2.6)	67.5 (6.4)
P54Ox	79.2 (48.3)	9.1 (5.6)	3.1 (1.9)	8.5 (5.2)	57.6 (13.7)	42.4 (18.7)

**Table 4:** Data for ash content, elemental analysis, and Boehm titration of the prepared ACs.

Sample	Ash Content (wt%)	Elemental Analysis				Titration (mmol g^−1^)				
						Acidity				Basicity
		C (wt%)	H (wt%)	N (wt%)	^1^ H/C	Strong	Medium	Weak	Total	Total
P54	1.2	84.2	1.4	0.3	0.20	0.55	0.07	1.21	1.83	0.00
P54red	1.3	91.0	0.6	0.6	0.08	0.29	0.18	0.68	1.15	0.23
P54Ox	1.1	72.8	1.4	1.6	0.23	1.26	0.42	1.19	2.87	0.05

^1^ Atomic ratio.

**Table 5:** Pt content in the prepared catalysts, as determined by ICP/OES.

Catalyst	Pt (wt%)	Catalyst	Pt (wt%)
Pt/P54-i	0.42	Pt/P54-i,ac	0.58
Pt/P54-w	0.65	Pt/P54-w,ac	0.67
Pt/P54Ox-w	1.00	Pt/P54Ox-w,ac	0.88
Pt/P54Red-w	0.79	Pt/P54Red-w,ac	0.73

**Table 6:** Average Pt particle sizes ($\bar{d}$) and contents of Pt in different oxidation states in selected catalysts.

Catalyst	$\bar{d}$ (nm)	^1^ Content (%)		
		Pt(0)	Pt(II)	Pt(IV)
Pt/P54-i	5.2	70.2	29.8	-
Pt/P54-w	3.9	56.0	44.0	-
Pt/P54-i,ac	3.0	33.1	28.2	38.7
Pt/P54Ox-w	6.8	73.6	26.4	-
Pt/P54Red-w,ac	2.7	51.8	48.2	-

^1^ Calculated considering the HR-XPS Pt 4*f* core level spectra.

**Table 7:** AIs of the products obtained in HDO tests.

Entry	Catalyst				HDO Test			AI
	Label	Preparation			Feedstock	Re-Reduction In Situ?	Reaction Time (h)	
		Support	^1^ HCl?	^2^ Impregnation				
1	None	-	-	-	Lauric acid	-	8	233.8
2	P54	P54	-	-	Lauric acid	-	8	220.8
3	Pt/P54-i	P54	No	IWI	Lauric acid	Yes	8	13.0
4	Pt/P54-w	P54	No	WI	Lauric acid	Yes	8	0.0
5	Pt/P54-w	P54	No	WI	Lauric acid	Yes	5	1.2
6	Pt/P54Ox-w	P54Ox	No	WI	Lauric acid	Yes	5	73.6
7	Pt/P54Red-w	P54Red	No	WI	Lauric acid	Yes	5	2.6
8	Pt/P54-i,ac	P54	Yes	IWI	Lauric acid	Yes	8	1.9
9	Pt/P54-w,ac	P54	Yes	WI	Lauric acid	Yes	8	0.0
10	Pt/P54-w,ac	P54	Yes	WI	Lauric acid	Yes	5	7.0
11	Pt/P54Ox-w,ac	P54Ox	Yes	WI	Lauric acid	Yes	5	81.0
12	Pt/P54Red-w,ac	P54Red	Yes	WI	Lauric acid	Yes	5	0.4
13	Pt/P54Red-w,ac	P54Red	Yes	WI	Lauric acid	No	5	0.0
14	Pt/P54Red-w,ac	P54Red	Yes	WI	Coconut oil	No	5	1.4
15	Pt/P54Red-w,ac	P54Red	Yes	WI	Coconut oil	No	7	0.0
16	Pt/P54Red-w,ac	P54Red	Yes	WI	Coconut oil	No	3	15.4
17	sulf-Ni,Mo/P54-w,4	P54	-	WI	Coconut oil	-	3	1.1
18	NiMoS/Al_2_O_3_	γ-Al_2_O_3_	-	-	Coconut oil	-	3	6.3
19	Pt/SAPO-11	SAPO-11	No	WI	Coconut oil	No	5	153.8

^1^ HCl added to the impregnating solution? ^2^ Impregnation methodology: IWI for incipient wetness impregnation; WI for wet impregnation.

## Data Availability

The original contributions presented in this study are included in the article. Further inquiries can be directed to the corresponding authors.

## Supplementary Materials

The following Supplementary Materials can be downloaded at: https://www.mdpi.com/article/10.3390/molecules30132862/s1: Figure S1. XPS survey spectra of the (**a**) unmodified AC P54 and the samples resulting from its (**b**) reductive thermal treatment in H_2_ atmosphere (P54Red) and (**c**) oxidative treatment in refluxing HNO_3_ solution (P54Ox); Figure S2. High-resolution XPS (**a**–**c**) C 1*s* and (**d**–**f**) O 1*s* core level spectra of the prepared activated carbons; Figure S3. Histograms of Pt particle size distribution of selected catalysts.

## Abbreviations

The following abbreviations are used in this manuscript:

AC	Activated carbon
BET	Brunauer–Emmett–Teller
DR	Dubinin–Radushkevich
EDX	Energy-dispersive X-ray
FEG	Field emission gun
FID	Flame ionization detector
GC	Gas chromatography
HAADF	High-angle annular dark-field
HDO	Hydrodeoxygenation
HEFA	Hydroprocessing of esters and fatty acids
HR	High-resolution
ICP	Inductively coupled plasma
IUPAC	International Union of Pure and Applied Chemistry
MS	Mass spectrometry
OES	Optical emission spectrometry
PSD	Pore size distribution
QSDFT	Quenched solid density functional theory
SAF	Sustainable aviation fuel
SSA	Specific surface area
STEM	Scanning transmission electron microscopy
SM	Supplementary Materials
TEM	Transmission electron microscopy
TPD	Temperature-programmed desorption
V_mic_	Micropores volume
V_mes_	Mesopores volume
XPS	X-ray photoelectron spectroscopy
XRD	X-ray diffraction
