# Supplementary material for: Activated Carbon-Supported Pt Catalysts Intended for the Hydroprocessing of Lipid Feedstocks: Effects of Support Surface Composition and Impregnation Protocol
Source: Molecules. 2025 Jul 4;30(13):2862. doi: 10.3390/molecules30132862 (PMC12251490; doi:10.3390/molecules30132862)
Supplement: Supplementary file 1 [file molecules-30-02862-s001.zip › molecules-3703218-supplementary.pdf]

molecules

MDPI

Article (Supplementary materials)

Activated Carbon-Supported Pt Catalysts Intended for the Hydroprocessing of Lipid Feedstocks: Effects of Support Surface Composition and Impregnation Protocol

Ruana D. Brandão [1,2], Antônio M. de Freitas Júnior [1,3], José J. Linares [1], Paulo A. Z. Suarez [1], Romulo C. Dutra [1], Jeremie Garnier [4], Myller S. Tonhá [4], Daniel Ballesteros-Plata [5], Enrique Rodríguez-Castellón [5,*] and Marcos J. Prauchner [1,*]

[1] Institute of Chemistry, University of Brasilia, Campus Darcy Ribeiro, Brasilia CEP 70904-970, DF, Brazil; ruanabrandao@gmail.com (R.D.B.); antoniomartinsdefreitasjunior@gmail.com (A.M.d.F.J.); joselinares@unb.br (J.J.L.); psuarez@unb.br (P.A.Z.S.); romulodutrac@gmail.com (R.C.D.)

[2] Department of Academic Areas, Federal Institute of Goiás, Rua 54, esq. com Rua 11, Parque Lago, Formosa CEP 73813-816, GO, Brazil

[3] Federal Institute of the North of Minas Gerais, Campus Arinos, Arinos CEP 38680-000, MG, Brazil

[4] Institute of Geosciences, University of Brasilia, Campus Darcy Ribeiro, Brasilia CEP 70910-900, DF, Brazil; garnier@unb.br (J.G.); myller@unb.br (M.S.T.)

[5] Department of Inorganic Chemistry, Crystallography and Mineralogy, Inter-University Institute of Research in Biorefineries I3B, Faculty of Sciences, University of Málaga, 29071 Malaga, Spain; daniel.ballesteros@uma.es

* Correspondence: castellon@uma.es (E.R.-C.); marcosjp@unb.br (M.J.P.)

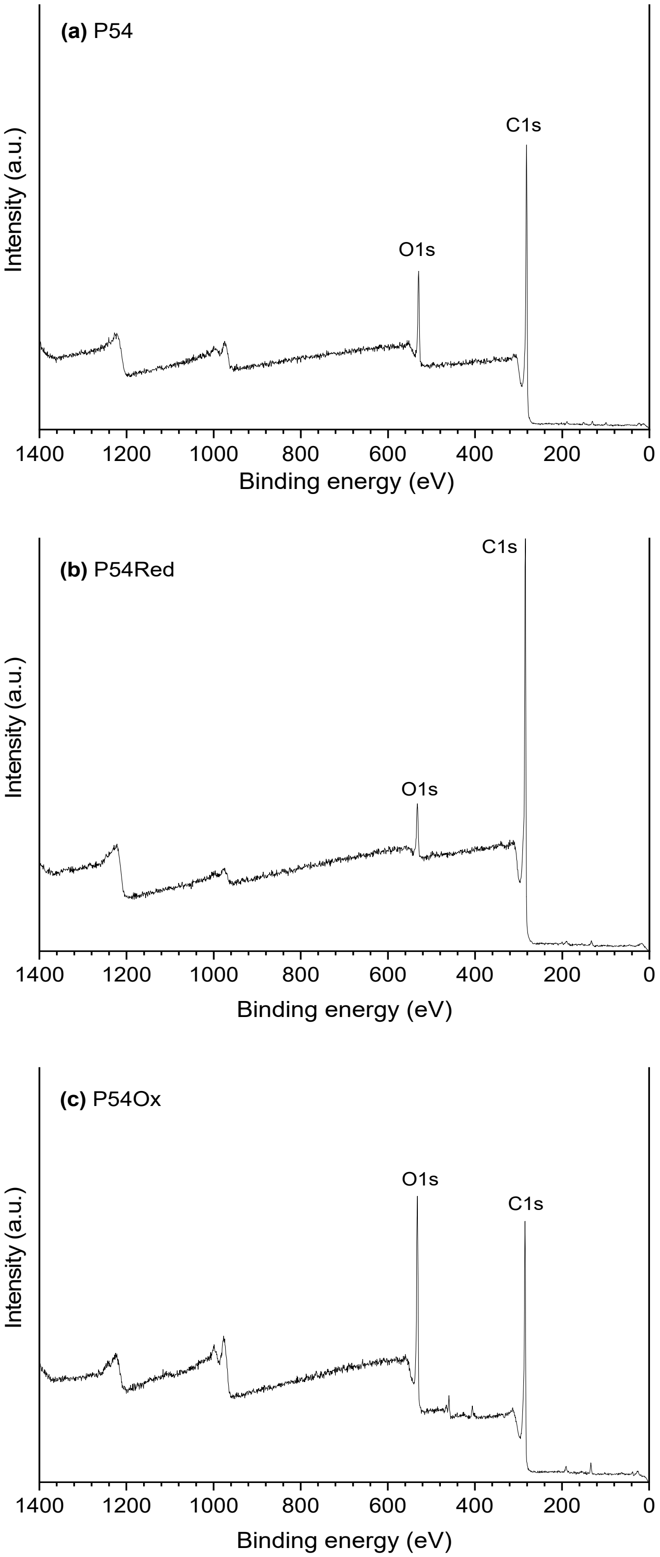

Figure S1. XPS survey spectra of the **(a)** unmodified AC P54 and the samples resulting from its **(b)** reductive thermal treatment in H_2 atmosphere (P54Red) and **(c)** oxidative treatment in refluxing HNO_3 solution (P54Ox).

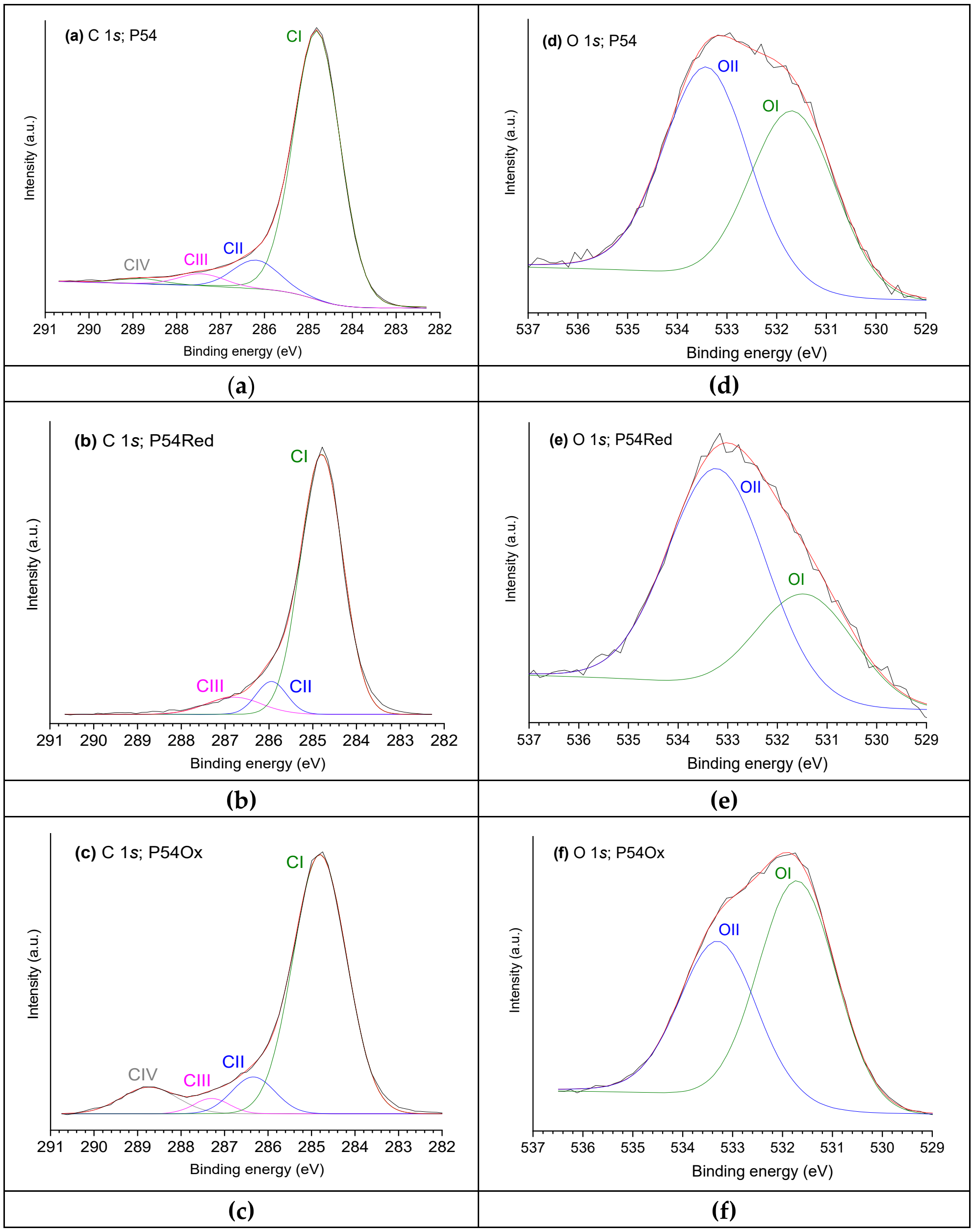

Figure S2. High-resolution XPS **(a–c)** C 1*s* and **(d–f)** O 1*s* core level spectra of the prepared activated carbons.

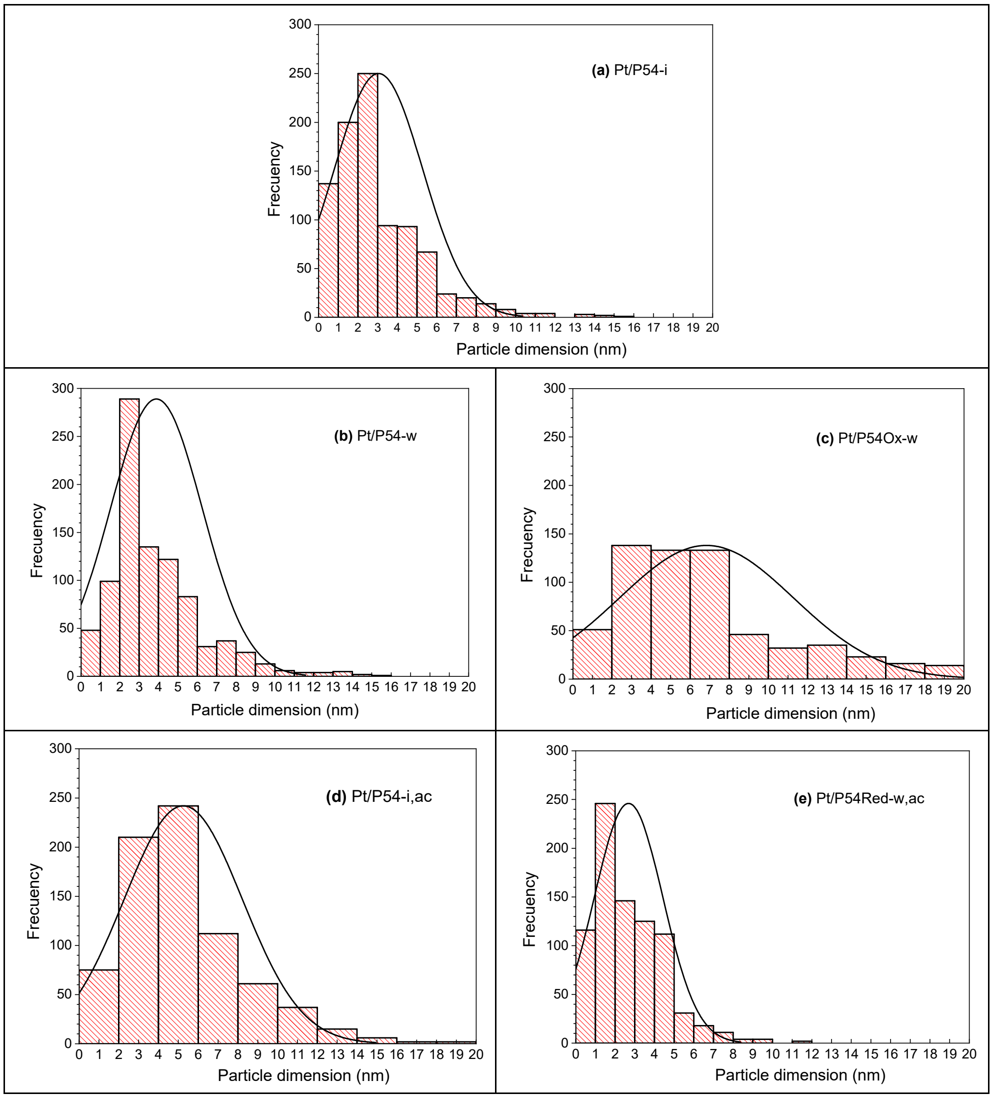

Figure S3. Histograms of Pt particle size distribution of selected catalysts.
